# Gender Stereotypes and Bias in Nursing: A Qualitative Study in Tanzania

**DOI:** 10.3390/nursrep15010014

**Published:** 2025-01-08

**Authors:** Racheal Mukoya Masibo, Golden M. Masika, Stephen M. Kibusi

**Affiliations:** School of Nursing and Public Health, The University of Dodoma, P.O. Box 259, Dodoma 41218, Tanzania; gmwakibo@yahoo.co.uk (G.M.M.); skibusi@gmail.com (S.M.K.)

**Keywords:** nursing, gender, stereotype, bias, discrimination

## Abstract

(1) The question addressed in this study is what kinds of stereotypes and biases of gender in nursing exist in Tanzania. This study aimed to investigate gender stereotypes and bias among healthcare providers and non-healthcare providers. (2) Methods: Qualitative descriptive design and data were collected from the Dar es Salaam region of Tanzania through a Focus Group Discussion approach. The qualitative content analysis was used to obtain themes the following themes. (3) Results: Three themes and eighteen subthemes emerged from this study. The first theme is role distribution based on nurse gender, its impact, and mitigating approaches for biased role distribution; the second theme is the different ways of addressing challenges in gender in nursing diversity; and the third theme is gender in nursing biases at the training institutions. (4) Conclusions: The bias and stereotypes about gender in nursing still exist in clinical areas and training institutions. Exercising professionalism in both settings remains a vital aspect of reducing bias. Moreover, role distribution should not be dominated by social roles of men and women in the community but rather should be based on competence and individual abilities.

## 1. Introduction

Even though the growth of the nursing profession is promising and its contribution to the implementation of Sustainable Development Goal (SDG) targets and the health and well-being of families and communities is undeniable [1,2], the profession has long been struggling with bias and stereotypes [3,4]. Throughout the 19th and 20th centuries, bias and gender stereotypes in nursing were predominant during the time nursing was considered a women’s profession with fewer men, but currently, there is a change in the trend of gender in nursing with a high influx of men in the profession [5].

Numerous studies have demonstrated how gender diversity in nursing is shifting. For example, an integrative review found that, despite the fact that men are under-represented in 104 countries, the recent campaigns in Australia and the UK have increased the number of men applying to study nursing by 50% [6]. In the meantime, a cross-sectional study found that although nursing has historically been seen as a woman’s profession, an increase in male applicants to nursing programs has been attributed to changes in the nature of the work and a greater knowledge of the field [7]. For instance, men made up 10,473 of the 52,256 nursing students enrolled in South Korea in 2019—above 20%—and this number is higher than the 9.7% growth in the percentage of male nursing students [7]. According to a qualitative study, more men are now entering the nursing field despite the fact that historically, the field was thought to be dominated by women [8]. The new data consistently refute the notion that nursing is solely a female-only profession [9]. Males are becoming more prevalent in nursing in a number of nations; in the US, the proportion of male nurses rose from 7% in 2006 to 9.6% in 2013 [10] and 12% in 2019 [11]. The proportion of male nurses in Singapore increased over time, rising from 8.2% in 2008 to 11.6% in 2020 [12]. Furthermore, the percentage of male nurses in New Zealand (8%), the UK (10.8%), and Australia (11.75%) is consistent with recent trends [11]. One may observe the evolving trend that gender shifts in nursing may be seen in educational attainment; in Slovenia, between 2010 and 2019, the average percentage of female nurses with a bachelor’s degree decreased from 93.83% to 88.66%, while the average percentage of male nurses with the same degree increased from 6.17% to 11.34% [13].

The rise in gender diversity in nursing influences changes in public policy, fosters professional development [14], increases job satisfaction [6], enhances staff interactions [15], and enhances patient care by giving patients the freedom to select their preferred gender [14]. Furthermore, gender diversity improves the profession and fosters a variety of attitudes and behaviors [15]. The Royal College of Nursing’s material shows how inevitable, accepted, and well-understood the necessity is for a diverse staff that reflects society [16]. Stereotypes and bias can hinder the achievement of diverse results in academic nursing and at all nursing levels, notwithstanding the importance of gender diversity in the field of nursing [17]. This supports the need to look at bias and gender stereotypes in nursing.

Numerous studies have demonstrated the patterns of gender stereotypes. For example, one study found that sexual harassment, unequal task and responsibility allocation, and a lack of support for career advancement are all examples of gender discrimination experienced by female nurses in the workplace [6] Male nurses, meanwhile, face several prejudices that cast doubt on their professional competence, their ability to control their sexual inclinations, and their masculinity [18]. In terms of bias, it is said that male nurses receive preferential treatment when it comes to career advancement, whereas female nurses are given preference for the majority of roles and possibilities [19]. There is doubt over the current state of affairs due to contradicting evidence regarding gender discrimination and preference in the nursing field. This necessitates conducting research to clear up any ambiguity and demonstrate the reality of the gender disparities that already exist. The reason for the confusion is that the descriptions of the studies that are now available are insufficient to demonstrate the pattern of gender stereotypes in nursing or to distinguish between male and female nurses who experience severe discrimination or are the targets of bias.

Even while the number of men entering the nursing field has encouraged scholars to study men in the field, a thorough qualitative analysis of the stereotypes surrounding males in the field has not yet been thoroughly investigated [8]. Although the number of male nurses entering the field is encouraging, a systematic fast review research found that stereotypes and gender prejudices continue to be major obstacles to the retention of male nurses [20]. Males who experience discrimination frequently decide to leave the nursing field and look for other possibilities [21]. Consequently, nursing professionals and faculty members should endeavor to comprehend their gender role stereotypes in order to lower the attrition rate among nursing students [7].

Stereotypes in nursing are generally categorized into stereotypes relating to the professionals’ gender and stereotypes relating to the profession itself [22]. This current study focuses on the stereotype of the professional gender, which is closely linked to a cultural construction of different types of behavior for men and women as a result of their sexual differentiation [22]. Bias, discrimination, and gender disparities in nursing continue to be problems that impede the best possible professional practice and professional development [23,24]. The gender trend in nursing is changing, with a large influx of men entering the field as opposed to earlier times when it was seen as a woman’s profession with fewer men [5]. Does the present rise in gender diversity in nursing bring prejudice and prejudices with it? What kind of prejudice and stereotypes might be applied in the wake of the shifts in nursing diversity?

Gender bias refers to the preference and preferential treatment of one gender over another [25]. Bias and stereotypes can be distinguished as follows: bias is a personal preference, like or dislike, especially when the tendency interferes with the ability to be impartial, unprejudiced, or objective, but a stereotype is a preconceived idea that attributes certain characteristics to all the members of a class or set [26].

This stereotype dates back to the early days of nursing when it was thought to be a vocation primarily for women. Florence Nightingale saw nursing as an extension of motherhood and believed that women were better suited for caring for others, and therefore, she automatically disregarded men who wanted to become professional nurses [13]. Consequently, the history of nursing and the lack of social acceptance of males in the field are the root causes of gender bias in nursing [15].

Like other nations, Tanzania had an under-represented male nursing workforce, with 14% of male nurses and 86% of female nurses [27]. A growing percentage of male nurses has been reported in the literature recently [28,29]. In 2015, for instance, the gender distribution of nurses shifted to 70% female and 30% male [30]. This comprehensive information can be found in our earlier paper [31]. Although there are benefits to more gender diversity in nursing, prejudice and stereotypes may also work against it [17]. There is a lack of information in the literature about bias and stereotypes in Tanzania, and it is unclear how stereotypes are structured. Thus, this study aimed to look into bias and gender stereotypes among Tanzanian patients, healthcare professionals who are not nurses, and nurses themselves. There were two goals for this study: (I) What kinds of gender biases exist in Tanzanian nursing? (II) How do patients, healthcare providers who are not nurses, and nurses feel about gender bias in nursing in Tanzania?

Regarding the problem statement, males quitting or planning to leave the profession are a barrier to the growth in gender diversity in nursing brought about by more men joining the field. Male nurses’ readiness to quit their employment in China varies from 11.1% to 85.7% [32]. Men are said to have left or plan to leave the field due to gender stereotypes [33,34]. Men quit their jobs because of job discontent brought on by stereotypes [21]. Even though men are quitting or planning to leave the nursing profession due to gender stereotypes, little is known about gender stereotypes and bias in Tanzania. Therefore, this study aimed to investigate gender stereotypes and bias among nurses, non-nurse healthcare providers, and patients in Tanzania.

Regarding the purpose statement, a qualitative descriptive study was conducted to explore gender stereotypes and biases existing in Tanzania among healthcare providers and non-healthcare providers. Through Focus Group Discussion (FGD) and the presence of an interview guide, the participants uncovered the pattern of gender stereotypes and bias in Tanzania.

Regarding this study’s significance, the description is based on empirical contributions and practical contributions. Since this current study is qualitative, the identified variables (themes and sub-themes) can be used to generate dimensions and items for data collection tools for quantitative studies. Moreover, it can assist researchers when developing interventional packages to mitigate stereotypes and biases. The findings will assist curriculum developers and curriculum reviewers in integrating essential elements/content beneficial to promote gender diversity. Moreover, this study will aid policymakers, administrators, leaders, managers, and guideline developers in considering strategies for eliminating bias in role distribution among nurses of different genders. This study will inform the nursing governing bodies, nursing associations, government, healthcare consumers, and healthcare stakeholders about the patterns of stereotypes and bias in clinical and training settings, which will help the invention of approaches to mitigate the problem.

## 2. Methods

### 2.1. Study Designs

This is a qualitative descriptive design to investigate the existing stereotypes and biases in gender in nursing among nurses, non-nurse healthcare providers, and patients in Tanzania. This qualitative descriptive design fits better in this current study than other qualitative approaches because it focuses on participants’ perceptions, attitudes, points of view, and experiences [35]. It focuses on the “who, what, and where” of experiences without deep theorization or recontextualization [36]. Compared to other qualitative approaches like ethnography, which reveals behavior and cultural aspects [37], phenomenology uncovers the experiential essence of phenomena [38], and grounded theory generates theories of social phenomena [39].

### 2.2. Study Setting

This study was carried out in the Dar es Salaam region of Tanzania due to its unique characteristics, including a national hospital and multiple regional hospitals, which facilitated the recruitment of a wide range of participants for the study.

### 2.3. Population

This study involved licensed nurses working at healthcare facilities, non-nurse healthcare providers (physicians, laboratory technicians, scientists, and pharmacists), and patients who attended healthcare facilities to seek medical attention. Nurses were recruited into the study because they are the victims of stereotypes and bias, but non-nurse healthcare providers like physicians, laboratory technicians, and pharmacists were considered in this current study because some of them are in managerial positions and are likely to cause bias, especially during role distribution, and others have observed experiences regarding bias and stereotypes among nurses. Non-healthcare providers like patients were considered as they are the witnesses of what male nurses and female nurses do. All participants were recruited from the hospital areas through a physical approach by researchers where they were asked to participate in the study.

### 2.4. Inclusion and Exclusion Criteria

Nurses, non-nurse healthcare providers, and patients who were fluent in Swahili’s native language or English were considered. Moreover, those present during the study, willing to participate, and assumed to have rich information were included in the study. Meanwhile, those who were very sick and unable to respond because of sickness were excluded from the study.

### 2.5. Sample Size and Sampling Procedures

The sample size was determined through the data saturation within each Focus Group Discussion (FGD). It was reached once no new issues on the explored area were coming out from participants. Regarding sampling procedures, nurses and non-nurse healthcare providers were recruited through purposive sampling, especially possessing rich information about the topic. The patients were recruited through convenience sampling techniques because only those available at the time of the study were considered.

### 2.6. Data Collection Procedures and Tools

The data exploring the existing bias of gender in nursing were collected by a principal investigator with one research assistant between August 2022 and January 2023. Three-day training of the research assistant was carried out, making her aware of the study’s aim, objectives, and expectations. She was trained on how to use an interview guide to collect data. Moreover, she was imparted with interview skills, especially on how to promote active participation of FGD members, avoid leading questions, and adhere to the interview guide. The interview guide developed by a principal investigator was utilized during interview sessions. This study was carried out at the selected four hospitals in Dar es Salaam. All participants were recruited and interviewed in the hospital settings. At the time of the interview session, the audio recorder was used to record the discussions. Furthermore, the interviewers had notebooks to note the non-verbal cues and areas of emphasis from participants. About data collection tools, the interview guides for nurses, non-nurse healthcare providers, and patients developed by a principal investigator were validated through face and content. The guide comprised two questions: (I) What can you say about the bias status in gender in nursing in Tanzania? (II) What would you suggest about the existing bias in gender in nursing?

### 2.7. Reflexivity and Bracketing

Two researchers (principal investigator and assistant) engaged in the data collection through the approach of Focus Group Discussion. The principal investigator was the female nurse, assistant lecturer, and Doctor of Philosophy (PhD) candidate focusing on nursing professionalism. The research assistant was a female nurse working in the clinical area, ward-in-charge, and holder of a master’s degree in nursing. Regarding authors, the team members had female and male academicians with diverse backgrounds in clinical expertise, professionalism, and public health. One team member, a female nurse (RMM), was an early career researcher (2 years of experience) and two of the team members, male nurses (SMK and GMM), had more than 10 years of experience in nursing qualitative research. These experiences contributed to the study’s conceptualization and research process at large. Even though the authors’ experiences and background seem to have repercussions on the study results, the identification, acknowledgement, evaluation, and self-consciousness about them assisted in resolving their effect [32]. There was no relationship established before the study’s commencement.

### 2.8. Data Analysis

After data collection, the data were transcribed verbatim and the recorded audio was converted into written text. The translation of the transcript from the native Swahili language into English was carried out by a linguist fluent in both the Swahili and English languages. The face validity of translated transcripts was performed by a principal investigator who is a nurse to ensure that the medical professional terms are used instead of lay words. Qualitative content analysis was used to analyze the data through the following steps: selecting the content to analyze, defining the units and categories of analysis, developing a set of coding rules, coding the text according to the rules, and analyzing the results and drawing conclusions [40]. Two researchers independently coded the data; each of the coders re-read each transcript and assigned codes. Once the coding template had been finalized independently, the coders met, each explained the organizational coding strategy, a and consensus was reached on how to label each discrete entity and how to categorize the type of comments that belong to each broad category [41].

### 2.9. Ethics Approval and Consent to Participate

This study’s ethical clearance letter was obtained from the University of Dodoma Institution Research Review Committee (IRREC) with reference number MA 84/261/02. Other permissions to conduct the study at the selected hospitals in Dar es Salaam were obtained from the Regional Administrative Secretary. Written and verbal informed consent was provided by each participant before being recruited into the study. Since this study recruited no one under 16 years old, there was no need to contact guardians to complete informed consent on behalf of those underage. The autonomy of participants was ensured by allowing participants to decide willingly to participate or to have the freedom to withdraw from the study at any time they wished to do so. The study had no form of harm to participants, rather, participants were asked to participate in the interview. Due to the long conversation of the Focus Group Discussion (FGD), the participants were provided with snacks and drinks to motivate and encourage them to be active. Privacy was maintained during the interview session by ensuring that the selected rooms for an interview could not allow people outside to hear the ongoing conversations. The confidentiality of patients’ information was maintained by keeping participant names anonymous, and the audiotaped recorder and transcripts were kept safe to avoid being accessed by non-researchers.

### 2.10. Trustworthiness

Credibility was ensured by involved data collectors who were professional nurses, holders of bachelor-level degrees and above, and trained. The involvement of different populations (nurses, non-nurse healthcare providers, and patients) and data triangulation across populations helped to establish credibility. Furthermore, member checks that involved a few participants to confirm the results also helped ensure credibility. Meanwhile, the FGDs that were conducted based on principles assisted in perpetuating credibility. Transferability, which involves the applicability of the findings to similar contexts, was achieved by detailed information provided within the methodological section, including data saturation, sampling procedure, techniques of data collection, and tool development and validation. Dependability was ensured through rigorous data collection techniques, procedures, and analyses that are well documented in the methodology section. Also, an expert as a reviewer of the study process was involved to ensure dependability. Confirmability to ensure findings would likely be repeatable by others was performed through bracketing and reflexivity. Moreover, the data were checked and rechecked throughout data collection and analysis to ensure that confirmability could be documented by a clear coding schema that identified the codes and patterns identified in the analysis.

## 3. Results

### 3.1. Study Characteristics

The data were separately collected from nurses, non-nurse healthcare providers, and patients. A total of 12 FGDs were conducted, with a total of 60 participants. Participants were recruited from four hospitals in Dar es Salaam and each population underwent a single FGD per hospital. The distribution and population demographic are as follows. A total of twenty-one nurses were recruited in all FGDs, with eleven females and ten males. The working experiences ranged from 2 to 10 years. Non-nurse healthcare providers (NN-HCPs) comprised sixteen participants of medical doctors, laboratory technicians, and pharmacists, with eight females and eight males, and working experiences ranging from 5 to over 22 years. Meanwhile, for patients, the data were collected from 23 participants (fourteen females and nine males). Refer to Table 1.

### 3.2. Themes

Through content analysis, three themes and eighteen subthemes emerged from this study. The following are the themes: ① role distribution based on nurse gender, impact, and mitigating approaches for biased role distribution; ② different ways of addressing challenges in gender in nursing diversity; and ③ gender in nursing biases at the training institutions. Throughout the document, the end of each quote has an abbreviation with FGD + number of population. For example, FGD1-N means the first Focus Group Discussion out of four, with N standing for the population of nurses. Moreover, FDG 3-NN-HCP means the third Focus Group Discussion, with NN-HCP standing for the population of non-nurse healthcare providers. Detailed information about themes and subthemes can be found in Table 2.

#### 3.2.1. Role Distribution Based on Nurse Gender, Impact, and Mitigating Approaches for Biased Role Distribution

**① Role distribution based on nurse gender, impact, and mitigating approaches for biased role distribution**

It has been reported by nurses, NN-HCPs, and patients in different FGDs that the role distribution in healthcare facilities is based on gender in nursing. Four subthemes emerged from this area, including the distribution of roles to male nurses at healthcare facilities, the distribution of roles to female nurses at healthcare facilities, the impact of biased role distribution on gender in nursing, and approaches to mitigate biased role distribution among gender in nursing.

#### 3.2.2. Distribution of Roles to Male Nurses at Healthcare Facilities

Nurses in FGDs said that it is assumed that some roles are more suitable for male nurses, which is why males occupy leadership positions; work in ICU or critical units; deliver care to serious patients; are assigned to physically demanding tasks or heavy lifting tasks; and have more hands-on patient care, specialized tasks, and technical work.

*“I have witnessed instances where certain nursing roles were associated more with a specific gender. For example, leadership positions were often occupied by male nurses, while female nurses were predominantly assigned to traditional caregiving roles”.*

*(FGD1-N)*

*“I see some biases because if you go to the ICU or critical units you will notice that the majority of the staff are male. There seems to be a bias in assuming that maybe certain roles require a specific gender, which can impact professional development opportunities”.*

*(FGD2-N)*

*“I’ve seen that serious patients are sometimes automatically assigned to male nurses, assuming they are better equipped to handle them. This perception creates a bias in the distribution of responsibilities”.*

*(FGD3-N)*

*“It’s not a lie as some or all of you normally see situations where male nurses are automatically assigned to handle physically demanding tasks, even if their skill set could contribute to other aspects of patient care”.*

*(FGD2-N)*

*“There were instances where me as a male nurse am automatically assigned heavy lifting tasks, assuming physical strength is my primary attribute”.*

*(FGD3-N)*

*“Most of the time male nurses are often allocated a lot of technical work than female counterparts”.*

*(FGD4-N)*

Male nurses, through role assignment, receive exceptional opportunities compared to female nurses.

*“I’ve noticed a bias in scheduling, where male nurses are often assigned more favourable shifts or rotations compared to their female counterparts. We have tried raising it in meetings but it seems to continue. It creates a disparity in work-life balance”.*

*(FGD3-N)*

*“For example, in meetings, workshops or seminars the male nurses were consistently chosen over equally qualified female counterparts”.*

*(FGD3-N)*

NN-HCPs in different FGDs identified bias in the role distribution among male nurses, such that male nurses are assigned to administrative or leadership decision-making roles, technical roles, and roles requiring physical strength and are allocated in male units.

*“In certain instances, I’ve observed some gender-based biases in role allocations among nursing teams. There have been cases where administrative or leadership roles might more frequently be offered to male nurses”.*

*(FGD1-NN-HCP)*

*“Male nurses are often directed towards roles that require physical strength, like patient lifting or emergency response cases”.*

*(FGD1-NN-HCP)*

*“I’ve noticed biases in role allocation as I have seen the majority of the in charges in male units are men”.*

*(FGD2-NN-HCP)*

*“I’ve observed some biases in the allocation of nursing roles”*

*“As I have worked for a considerable long time interacting and directing nurses, I know in some cases, decision-making roles or positions involving technical aspects were seemingly more directed towards male nurses”.*

*(FGD2-NN-HCP)*

*“I haven’t noticed any bias but I tend to think that in a ward with both male and female nurses and there is a need to lift or carry a patient, the eyes will be directed to a male nurse rather than a female nurse”.*

*(FGD4-NN-HCP)*

*“Male nurses are mostly given the leadership roles unlike the female nurses”.*

*(FGD4-NN-HCP)*

The patients in FGDs supported that there are roles that seem specific for male nurses in clinical areas, such as physical strength tasks, leadership, administration of medicine, assisting mobility exercises, turning patients, technical tasks, and practical tasks. Furthermore, male nurses are allocated to work in male units.

*“I can share from what I have observed since am not a nurse. I tend to think that the roles requiring more physical strength were often assigned to male nurses, regardless of their skills or expertise in other areas”.*

*(FGD1-C)*

*“In my experience, male nurses are sometimes assumed to be more commanding, which I tend to think has led to their frequent assignment to leadership roles. Female nurses, even those with strong leadership skills, are sometimes overlooked for these positions”.*

*(FGD1-C)*

*“I expect a lot of male nurses to be leaders and managers”.*

*(FGD2-C)*

*“I can share a recent experience when I went to stay with my father in the hospital, I noticed that male nurses were always giving out drugs and turning the patients who could not stand up”.*

*(FGD3-C)*

*“I observed a similar pattern during a hospital stay. Male nurses were often called upon for more practical tasks, even if a female nurse might have been equally qualified. It gave the impression that certain responsibilities were for men, rather than the nurse’s expertise”.*

*(FGD3-C)*

*“Male nurses were sometimes given male patients assuming it might be more comfortable for them”.*

*(FGD3-C)*

*“I once was admitted in a male ward and the majority of the nurses were male”.*

*(FGD3-C)*

*“I was once in the receiving unit waiting to see a doctor and a patient arrived tied up and I observed male nurses tasked to handle the patient”.*

*(FGD3-C)*

*“In a previous hospital stay, a male nurse was automatically assigned more physically demanding tasks, like lifting patients and giving medications”.*

*(FGD4-C)*

*“Male nurses are seen as more suited for technical tasks”.*

*(FGD4-C)*

#### 3.2.3. Distribution of Roles to Female Nurses at Healthcare Facilities

Nurses in FGDs revealed that in the current practice within healthcare facilities, there seem to be specific roles directed to female nurses, such as paperwork and documentation tasks, bedside care/caregiving/direct patient care, and administrative tasks.

*“There’s a tendency to assign more paperwork and documentation tasks to female nurses”.*

*(FGD3-N)*

*“Female nurses were predominantly assigned to bedside care”.*

*(FGD3-N)*

*“Female nurses were predominantly assigned to traditional caregiving”.*

*(FGD1-N)*

*“Female nurses are often assigned direct patient care”.*

*(FGD4-N)*

*“I’ve encountered situations where we as female nurses were assumed to take on more administrative tasks, even when they were equally capable of handling clinical responsibilities”.*

*(FGD4-N)*

*“In my experience, there’s been a tendency to assign certain tasks, like administrative duties, more often to female nurses. It creates an imbalance in responsibilities and may reinforce traditional gender roles”.*

*(FGD1-N)*

The bias in the role distribution to female nurses was discussed by NN-HCPs, who revealed that female nurses are assigned to caregiving roles/bedside roles, nurturing roles, and some administrative tasks. They are more often appointed to be in charge of female units and are assigned to work in pediatric units, maternity units, and labor wards.

*“Female nurses are more often allocated to tasks involving direct patient care or nurturing roles”.*

*(FGD1-NN-HCP)*

*“It’s hard to point out in the nursing field but I too have witnessed instances where gender-based biases influenced the allocation of nursing roles. For example, there are cases where female nurses were more frequently assigned to some administrative tasks”.*

*(FGD1-NN-HCP)*

*“I’ve noticed biases in role allocation as I have seen in the pediatric and female units are women”.*

*(FGD2-NN-HCP)*

*“I’ve observed some biases in the allocation of nursing roles where caregiving or bedside roles were often allocated to female nurses”.*

*(FGD2-NN-HCP)*

*“I tend to see the majority of females in either pediatric or maternity units”.*

*(FGD3-NN-HCP)*

*“I think there are some situations most women in this hospital are in charge”.*

*(FGD3-NN-HCP)*

*“I think I have noticed that the majority of nurses in the labour ward are female nurses which might be perceived as more suitable for one gender over another”.*

*(FGD4-NN-HCP)*

Further, the patients identified specific roles that seem distributed to female nurses in clinical areas, such as caring, nurturing, documentation, taking vital signs, making beds, assisting in washing patients, delivering health education to patients, and communicating demanding tasks. Moreover, female nurses are allocated to pediatric wards, maternity wards, and other units with female patients. They are also assigned to multitask and handle complex patient interactions.

*“On the other hand, female nurses I think are directed towards more caring roles and nurturing”.*

*(FGD1-C)*

*“Female nurses seemed to be given more of the caregiving like taking temperatures and sitting in their office filling up books”.*

*(FGD2-C)*

*“In my experience, I have seen instances where male nurses are not assigned in the maternity ward. It’s as if there’s an automatic assumption that women are more suitable for these areas”.*

*(FGD1-C)*

*“I observed that female nurses were often assigned to discuss topics like prenatal care or family planning, how to breastfeed, and washing the babies. I never saw a male nurse offering the education even though I could see some of them moving around”.*

*(FGD3-C)*

*“Female nurses were directed towards female patients, which might not always be the patient preferences”.*

*(FGD3-C)*

*“I have visited my wife when she was admitted to the female ward and most nurses I saw were females”.*

*(FGD3-C)*

*“I was once in the receiving unit waiting to see a doctor and a patient arrived tied up and I observed female nurses were told to fill the forms”.*

*(FGD3-C)*

*“Female nurses seemed to make beds and also assist in washing us”.*

*(FGD4-C)*

*“There seems to be an assumption that female nurses are better at multitasking and handling complex patient interactions”.*

*(FGD4-C)*

*“I’ve seen female nurses being directed more towards paperwork, documentation, and communication tasks, even if they were perfectly capable of handling the physical aspects of patient care”.*

*(FGD4-C)*

***Impact of biased role distribution on gender in nursing***

The patients in different FGDs identified that the biased role distribution based on nursing gender has a negative outcome for nurses themselves. It can create a sense of frustration, inequality, lack of motivation, and stigmatization among the nursing staff. Biased role distribution perpetuates an unequal distribution of workload, reinforces outdated stereotypes about men and women, and makes some nurses feel that they need to conform to certain expectations based on gender rather than being recognized for their unique skills.

*“It can create a sense of frustration and inequality among the nursing staff as female nurses might feel overlooked, while male nurses may feel stigmatized as it seems to follow gender stereotypes”.*

*(FGD1-C)*

*“It could create a sense of frustration among the nurses. It might also lead to a lack of motivation to nurses”.*

*(FGD3-C)*

*“It can also create tension within the team as some nurses may feel that they need to conform to certain expectations based on gender rather than being recognized for their unique skills”.*

*(FGD1-C)*

*“It creates an unequal distribution of workload and can lead to stress especially if certain tasks are consistently assigned based on gender rather than skills. Maybe it might be the reason some nurses are never happy with their roles”.*

*(FGD4-C)*

*“I think it also reinforces outdated stereotypes about what men and women are “supposed” to be good at in the healthcare field, which doesn’t reflect the reality of the skills among nurses”.*

*(FGD4-C)*

***Approaches to mitigate biased role distribution between genders in nursing***

Several strategies have been proposed by nurses and NN-HCPs, including transparency during role distribution; establishing role qualifications; having objectives, criteria, and guidelines; making resources available to both genders; leader initiatives; training; open communication; and fairness.

### 3.3. Mitigating Biased Role Distribution Through Transparency

Most nurses in FGDs suggested that biased role distribution can be addressed by exercising transparency during role distribution.

*“I think promoting transparency in the decision-making process for role assignments can be crucial”.*

*(FGD2-N)*

Similarly, NN-HCPs emphasized the demanding transparency process regarding role distribution.

*“Implementing a transparent and merit-based system for role allocations”.*

*(FGD1-NN-HCP)*

*“Establish clear and transparent processes for assigning roles”.*

*(FGD1-NN-HCP)*

Similarly, patients mentioned that during role distribution of roles to nurses, transparency should be exercised.

*“I think nurses should engage in open and transparent communication with us as patients”.*

*(FGD1-C)*

### 3.4. Mitigating Biased Role Distribution Through Open Communication

NN-HCPs indicated that open communication during role distribution will aid in avoiding bias.

*“Foster an environment where nurses can openly discuss concerns related to role allocations for continuous improvement and fairness in task assignments”.*

*(FGD3-NN-HCP)*

*“I think communication and teamwork is important”.*

*(FGD3-NN-HCP)*

The same can be applied to the patients in FGDs, who indicated that open communication is vital in the distribution of roles among nurses, as it avoids distribution inequalities.

*“I would also like to emphasize the need for clear communication”.*

*(FGD4-C)*

### 3.5. Mitigating Biased Role Distribution Through Fairness

It was reported by NN-HCPs that exercising fairness in clinical areas during role distribution is important in abolishing the existing biases.

*“Ensure fairness in nursing role distribution is possible if we adhere to a merit-based system”.*

*(FGD4-NN-HCP)*

*“Periodically assess role allocations to ensure fairness and equality”.*

*(FGD4-NN-HCP)*

Similarly, the patients reported that fairness in role distribution is very important to avoid biases.

*“I think a fair mix of both genders in leadership positions is important which might bring an understanding of individual strengths and not have a lot of men in those posts”.*

*(FGD3-C)*

### 3.6. Mitigating Biased Role Distribution Through Establishing Role Qualifications

Most of the nurses in FGDs mentioned that the distribution of roles should be based on the qualifications of nurses and not on their gender.

*“Ensuring that qualifications and skills are the primary criteria can help eliminate biases”.*

*(FGD1-N)*

In the same way, NN-HCPs insisted that roles in nursing should be assigned based on competencies and interests. They should be distributed based on qualifications, skills, and experience rather than gender.

*“I also think that roles in nursing should be assigned based on competencies and interests rather than assumptions about gender capabilities helps in avoiding biases in role allocations”.*

*(FGD1-NN-HCP)*

*“Ensuring they’re based on qualifications, skills, and experience rather than gender”.*

*(FGD1-NN-HCP)*

*“Assigning roles based on skills, qualifications, and experience rather than gender assumptions can eliminate biases and if unfortunately, one gender is more qualified then we accept the reality and embrace the system”.*

*(FGD3-NN-HCP)*

Consistently, the patients responded that it is noteworthy to distribute roles to nurses based on individual abilities, experiences, and skills, rather than gender.

*“It’s not just about personal preferences; it’s about recognizing and utilizing everyone’s skills to provide the best care possible”.*

*(FGD4-C)*

### 3.7. Mitigating Biased Role Distribution Through Having Objective, Criteria, Guidelines, and Policy

Other participating nurses recommended a demand for the objective, criteria, and guidelines to help in the decision to assign roles to nurses.

*“Implementing clear, objective, and criteria for role assignments, along with regular evaluations to ensure fairness could be beneficial”.*

*(FGD1-N)*

*“Establishing clear and unbiased criteria for role assignments could be crucial”.*

*(FGD3-N)*

*“Establishing clear and objective criteria for role assignments can minimize the influence of subjective factors”.*

*(FGD4-N)*

*“Having clear guidelines and criteria for role assignments can be beneficial”.*

*(FGD3-N)*

NN-HCPs added that policies related to role distribution should be available to avoid human preferences during role distribution.

*“Policies should be put in place to focus on skills, qualifications, and experiences rather than assumptions based on gender”.*

*(FGD3-NN-HCP)*

Patients in FGDs added that having an established guide for distributing roles is the key to avoiding bias.

*“I believe clear guidelines that focus on individual abilities and experiences rather than gender”.*

*(FGD3-C)*

### 3.8. Mitigating Biased Role Distribution by Making Resources Available to Both Gender

It has been mentioned that the equal distribution of resources to both genders of nurses can be a factor in minimizing the triggers of biased role distributions.

*“Ensures that both male and female nurses have access to the resources needed for advancement and going to study”.*

*(FGD2-N)*

### 3.9. Mitigating Biased Role Distribution Through Leaders’ Initiatives

Nurses in FGDs stated that leaders at healthcare facilities are key players in ensuring that bias in role distribution is not practiced. Leaders should be involved in assigning roles based on qualification, embracing diversity in nursing, and often reviewing and evaluating role distribution.

*“Make sure as leaders that individuals are selected based on their skills and qualifications rather than gender in cases of assigning roles”.*

*(FGD2-N)*

*“The management should recognize and appreciate the diverse skills each nurse has”.*

*(FGD4-N)*

*“The leaders should regularly review and evaluate the distribution of roles to ensure fairness is also essential”.*

*(FGD4-N)*

The involvement of leaders in the mitigation of role distribution bias in gender in nursing is mentioned by NN-HCPs, who stated that leaders should conduct regular evaluations to check whether roles are distributed based on an individual’s ability.

*“As leaders, we need to periodically evaluate role assignments and responsibilities to ensure fairness and equity and rectify any biases that might exist”.*

*(FGD2-NN-HCP)*

*“Periodically assess role allocations to ensure fairness and equality. Hold decision-makers accountable for maintaining an equitable distribution of nursing roles”.*

*(FGD4-NN-HCP)*

### 3.10. Mitigating Biased Role Distribution Through Training

Training has been mentioned by most nurses in different FGDs as a means to mitigate the bias in role distribution.

*“Conducting regular training sessions on unbiased role assignments and promoting diversity in leadership can help create a more inclusive environment”.*

*(FGD4-N)*

*“The need for training and education on unconscious biases can help in recognizing and rectifying these issues in role assignments”.*

*(FGD4-N)*

*“I think regular team training sessions focusing on diversity, unconscious bias, and effective communication could help”.*

*(FGD3-N)*

*“I think regular training could also help create awareness among healthcare providers and reduce biases”.*

*(FGD3-N)*

NN-HCPs have also suggested the need to increase awareness, which might help avoid any emerging bias during role distribution.

*“There is a need to increase awareness that can help in challenging and eliminating biases in role allocations”.*

*(FGD2-NN-HCP)*

The patients agree with NN-HCPs and nurses by saying that nurses need to develop their careers and attend training to acquire the competencies required for certain roles.

*“Undergoing regular training sessions as well to have all-round skills”.*

*(FGD3-C)*

*“I believe education is key to keep them aware and informed”.*

*(FGD4-C)*

### 3.11. Mitigating Biased Role Distribution by Granting Equal Opportunities

Many NN-HCPs in different FGDs discussed that both male and female nurses should receive equal opportunities to advance their careers to obtain the required competencies.

*“I also concur with the need for providing equal opportunities for skill development and career advancement”.*

*(FGD1-NN-HCP)*

*“Ensuring continuous professional development allows everyone to acquire the expertise needed for different roles, reducing the impact of gender biases”.*

*(FGD4-NN-HCP)*

*“Provide equal opportunities for career advancement and professional development to all nurses, irrespective of gender”.*

*(FGD2-NN-HCP)*

Additionally, NN-HCPs reported that nurses of all genders need to have equal opportunities in leadership. They emphasized the need to support all genders to participate in leadership.

*“Support both genders in pursuing diverse roles and leadership positions within nursing”.*

*(FGD1-NN-HCP)*

*“We should encourage diversity in leadership roles within nursing because some of us here are decision-makers in selecting leaders. We need to promote and support individuals based on merit and capability rather than adhering to gender stereotypes”.*

*(FGD2NN-HCP)*

*“There is a need to promote and support leadership opportunities for nurses of all genders so that certain posts should not be directed to certain nurse genders”.*

*(FGD3-NN-HCP)*

### 3.12. Mitigating Biased Role Distribution Through Valuing Diversity and Inclusiveness

The NN-HCPs reported that when there is a culture of valuing the diversity of gender in nursing at healthcare facilities, it is likely that even role distribution will not be biased.

*“Create a work environment that values diversity and inclusivity, where all nurses feel empowered to pursue various roles without gender-related limitations”.*

*(FGD2-NN-HCP)*

### 3.13. Other Mitigating Strategies

Patients mentioned other strategies that may avoid bias in role distribution between genders in nurses. They emphasized that role distributors should seek opinions, identify the existing biases, and rectify them. Additionally, other strategies included having an award each year to recognize a male and female nurse who performed well, avoiding calling a nurse “sister” as it is biased in itself because a male nurse cannot be called “sister”, developing a culture of respect within healthcare organizations, and considering feedback from patients

*“To identify and rectify any biases that may mistakably influence the assignment of nursing roles for improvement”.*

*(FGD1-C)*

*“Seek our opinions as you are doing now maybe it might help them adjust their practices accordingly”.*

*(FGD3-C)*

*“I’d add that having an award each year to recognize a male and female nurse who has performed well”.*

*(FGD3-C)*

*“I think the education system is trying not to be biased but the continuous calling a nurse “sister” is bias in itself because you can’t call a male nurse sister”.*

*(FGD3-C)*

*“I think including our feedback as patients is also important because we also notice and experience a lot”.*

*(FGD4-C)*

*“I’d add that development a culture of respect within healthcare organizations is important. as it naturally translates into more personalized and patient-centered care”.*

*(FGD4-C)*

**②** **Different ways of addressing challenges in gender in nursing diversity**

Several approaches were suggested by nurses, NN-HCPs, and patients to overcome bias in gender diversity, including training and mentorship, leaders’ efforts, open communication, mutual respect, recognizing and valuing team member skills and contributions, valuing the diversity of gender in nursing, collaboration, prioritizing the needs and preferences of patients above all else, and equitable treatment for all team members.

***Addressing bias in gender in nursing diversity through training and mentorship***

The majority of nurses in FGDs identified training and mentorship as an effective means to address the existing and perpetuating bias in gender in nursing.

*“I think education and awareness programs within healthcare institutions can play a crucial role”.*

*(FGD-1)*

*“It’s essential for nurses to continuously learn and adapt their practices”.*

*(FGD2-N)*

*“Training programs could be designed to promote and accommodate both male and female nurses without feeling that you are in the wrong profession”.*

*(FGD2-N)*

*“My emphasis still lies on regular training and awareness programs for healthcare providers can help sensitize them to gender biases”.*

*(FGD2-N)*

*“I think regular team training sessions focusing on diversity, unconscious bias, and effective communication could help”.*

*(FGD3-N)*

*“Having a mentorship program could also be beneficial whereby nurses are paired up with mentors based on skills and potential rather than gender can contribute to a more equitable distribution of responsibilities”.*

*(FGD2-N)*

*“I also think we need to be paired with nurses with mentors from different genders who might offer insights and support, creating opportunities for skill development and understanding diverse perspectives”.*

*(FGD3-N)*

Through training, people become aware that optimal performance is not connected to gender in nursing, but rather, it is about competence.

*“I believe ongoing diversity and inclusion training for healthcare teams can enhance awareness and sensitivity to different identities”.*

*(FGD1-N)*

*“We also need to understand that each gender brings unique strengths”.*

*(FGD3-N)*

It is emphasized that everyone at the clinical facilities should understand the importance of equal distribution of roles.

*“Ensuring that everyone, from leadership to frontline staff, understands the importance of equitable role distribution can lead to positive changes”.*

*(FGD1-N)*

When training is delivered, it promotes respect for everyone and creates a supportive environment in which everyone is important regardless of gender.

*“Regular training on diversity and inclusion can contribute to creating a supportive environment for everyone is also important”.*

*(FGD1-N)*

NN-HCPs also mentioned that it is inevitable to deliver training and education to address gender sensitivity. Therefore, there is a need to provide opportunities for ongoing education and training.

*“Provide opportunities for continuous learning and skill development, focusing on diversity training, communication strategies, and cultural competency to better understand and cater to the needs of diverse patient populations”.*

*(FGD1-NN-HCP)*

*“Continuously invest in training and education that addresses gender sensitivity”.*

*(FGD2-NN-HCP)*

*“Encourage ongoing education and training that address gender sensitivity”.*

*(FGD4-NN-HCP)*

Patients consistently mentioned that education is crucial for eliminating gender bias in nursing.

*“Education is important”.*

*(FGD1-C)*

***Addressing bias in gender in nursing diversity through leaders’ efforts***

Leaders seem to be key players in the mitigation of bias in gender in nursing. It is recommended that leaders should undergo training to become aware of what exists and how to address problems.

*“Encouraging diversity training for leadership and decision-makers can be impactful. As It can raise awareness about unconscious biases and foster a culture”.*

*(FGD3-N)*

Leaders need to lead by example by discouraging the habits fostering the growth of bias in the diversity of gender in nursing.

*“We need to encourage individuals to lead by example in challenging gender-related stereotypes and promoting inclusive practices”.*

*(FGD3-N)*

*“It is important that leadership values equality”.*

*(FGD3-N)*

Leaders need to adopt the habit of revisiting the role assignment to nurses to be able to identify and address any emerging bias.

*“Regular assessments of role assignments and promotions can help identify and rectify any gender-based biases”.*

*(FGD1-N)*

Patients in the same way put forth that leaders have the potential to ensure that there is a positive elements of team dynamics.

*“Leaders within nursing teams should positively prompt the team’s dynamics”.*

*(FGD1-C)*

***Addressing bias in gender in nursing diversity through open communication***

Nurses in FGDs reported that open communication among the team is essential as it enables nurses to discuss all issues related to gender and calls for the need to create an environment for nurses to share what they feel and perceive about gender diversity.

*“Fostering open communication and creating spaces for team members to share their experiences and perspectives can promote understanding and collaboration. It’s about embracing diversity as a strength within the team”.*

*(FGD1-N)*

*“Creating a supportive environment where team members can openly discuss and address any concerns related to gender or other biases is essential”.*

*(FGD3-N)*

*“I believe effective communication among the nursing team is the key, it creates a collaborative environment that directly benefits patient care”.*

*(FGD1-N)*

*“I believe fostering a culture of respect and open communication within the team is crucial”.*

*(FGD2-N)*

*“I think we need to create an environment where everyone feels comfortable sharing their thoughts and experiences without fear of judgment”.*

*(FGD3-N)*

NN-HCPs reported similar issues and exhibited that open dialogue among team members is important as it allows them to discuss challenges related to their gender identity.

*“I believe that encouraging open communication within nursing teams is crucial where everyone will feel comfortable sharing concerns or suggestions can strengthen teamwork and improve patient care outcomes”.*

*(FGD4-NN-HCP)*

*“Encourage open dialogue and communication among team members, promoting an environment where everyone feels heard and valued”.*

*(FGD2-NN-HCP)*

*“We need to encourage open communication among nursing teams, fostering an environment where ideas and concerns can be freely discussed”.*

*(FGD1-NN-HCP)*

On the other hand, patients in FGD reported that having open communication at healthcare facilities will reduce or mitigate the bias in gender diversity.

*“I believe communication is also important”.*

*(FGD1-C)*

***Addressing bias in gender in nursing diversity through mutual respect***

Nurses said that when nurses at healthcare facilities embrace the culture of respecting each other regardless of gender, it enables everyone to feel that they belong to the team.

*“I think is all about fostering a culture of respect and inclusivity”.*

*(FGD3-N)*

*“Maybe I can also say that creating a culture of mutual respect and understanding among nursing teams is essential”.*

*(FGD2-N)*

*“I believe mutual respect among the nursing team is the key. When everyone values each other’s contributions”.*

*(FGD1-N)*

NN-HCPs in FGDs emphasized further that the biased gender diversity can be resolved once diverse perspectives are respected by everyone at a healthcare facility. Also, every nurse needs to feel respected and valued regardless of gender.

*“It’s important to acknowledge that each individual, regardless of gender, brings unique qualities to patient care. Valuing and respecting these differences create a more inclusive and collaborative healthcare environment”.*

*(FGD1-NN-HCP)*

*“The nurses regardless of gender should feel valued, respected, and empowered to contribute their unique skills and perspectives”.*

*(FGD1-NN-HCP)*

*“Nurture an inclusive environment that values and respects diverse perspectives, experiences, and identities within the nursing team”.*

*(FGD2-NN-HCP)*

*“Foster an inclusive environment where every nurse, regardless of gender identity, feels respected, valued, and empowered to contribute their unique skills and expertise to patient care”.*

*(FGD3-NN-HCP)*

Patients also reported that mutual respect among nurses is crucial to avoid bias over gender in nursing.

*“I think communication and mutual respect within nursing teams is crucial regardless of gender identity, which will improve patient care”.*

*(FGD4-C)*

***Addressing bias in gender in nursing diversity by recognizing and valuing team member skills and contributions***

Most nurses in FGDs put forward that each nurse’s strengths and contributions need to be acknowledged. It must be assumed that gender in nursing has nothing to do with the impact on a nurse’s performance, but rather, their skills are necessary.

*“I also think that it’s also about recognizing and valuing each team member’s strengths”.*

*(FGD3-N)*

*“It has heightened my expectations for a nursing practice that actively addresses these biases, creating a supportive environment where each nurse is recognized for their skills and contributions rather than gender”.*

*(FGD2-N)*

*“It emphasizes the need for a shift towards recognizing and valuing competence and skills over traditional gender roles”.*

*(FGD1-N)*

*“I think a culture of encouraging collaboration and recognizing individual strengths rather than assuming based on gender can significantly impact teamwork”.*

*(FGD3-N)*

*“It has reinforced the importance of fostering an inclusive nursing culture. It highlights the need for nurses to be evaluated based on their capabilities rather than predefined gender roles, ensuring fair treatment and opportunities for everyone”.*

*(FGD1-N)*

Along the same lines, NN-HCPs disclosed that it is appreciated to acknowledge nurses’ strengths, clinical competence, and unique qualities instead of looking at gender.

*“It’s about fostering an environment where nurses, regardless of gender, can excel based on their strengths”.*

*(FGD3-NN-HCP)*

***Value the diversity of gender in nursing***

NN-HCPs indicated that embracing diversity in nursing and recognizing varied perspectives and experiences of each gender has a positive effect on patient care and team collaboration.

*“Recognizing and understanding these differences is crucial for effective collaboration”.*

*(FGD1-NN-HCP)*

*“Nurture a culture that values and celebrates diversity among nurses, recognizing that varied perspectives and experiences contribute to a comprehensive approach to patient care”.*

*(FGD3-NN-HCP)*

***Addressing bias in gender in nursing diversity through collaboration***

NN-HCPs responded that one strategy for eliminating bias in gender in nursing can be through establishing a culture of collaboration among team members.

*“Foster a culture of teamwork and collaboration, where nurses support each other’s strengths and work cohesively towards common goals, centred on providing excellent patient care”.*

*(FGD3-NN-HCP)*

*“Strengthen teamwork by emphasizing collaboration”.*

*(FGD4-HCP)*

Patients in FGDs also emphasized that when there is teamwork at the healthcare facilities, it prevents the growth of bias in gender in nursing.

*“Teamwork among nurses is important”.*

*(FGD4-C)*

***Prioritizing the needs and preferences of patients above all else***

It was unzipped by NN-HCPs that when nurses put forward the demands of patients, it might lessen the gender bias in nursing.

*“Maintain a patient-centred approach, prioritizing the needs and preferences of patients above all else”.*

*(FGD4NN-HCP)*

***Equitable treatment for all team members***

NN-HCPs emphasized that there should be equal treatment of nurses from both genders and equal distribution of opportunities for them.

*“There is a need to promote a culture that emphasizes merit-based opportunities and equitable treatment for all team members”.*

*(FGD1-NN-HCP)*

*“Actively advocate for equitable opportunities”.*

*(FGD3-NN-HCP)*

**③** **Gender in nursing biases at the training institutions**

Nurses, NN-HCPs, and patients indicated that the bias of gender in nursing is prevalent at the training institutions, as male nursing students are more favored in receiving opportunities than female nursing students. The process of imparting nursing skills to male nursing students is somehow different compared to how female nursing students acquire skills. Participants’ discussion can be categorized as follows: addressing teaching methods encouraging gender bias in nursing at the training institutions, the existence of unequal opportunities for gender in nursing at the training institution, the bias against male nurse students at the training institutions, the bias against female nurse students at the training institutions, and addressing gender bias at the training institutions.

***Teaching methods encouraging gender in nursing bias at training institutions***

NN-HCPs communicated that the teaching process including approaches demonstrates bias in training institutions. The scenarios, case studies, role plays, and other teaching methods reflect the existing gender stereotypes in nursing.

*“There have been instances where certain teaching methodologies or expectations might have exhibited gender-based biases”.*

*(FGD1-NN-HCP)*

*“In my experience, there have been instances where gender-based biases surfaced in nursing education. Some teaching methodologies or curricula might unintentionally reinforce gender stereotypes. For example, certain clinical scenarios or case studies might stereotypically depict gender roles, potentially influencing students’ perceptions”*

*(FGD2-NN-HCP)*

*“There might be instances where role-playing exercises or teaching materials inadvertently portray gender biases. These biases could impact students’ perceptions of the roles and capabilities of nurses based on gender”.*

*(FGD2-NN-HCP)*

*“I’ve seen instances where teaching methods or discussions inadvertently reflected gender biases. This might affect how students perceive nursing roles and responsibilities based on gender stereotypes presented during their education”.*

*(FGD3-NN-HCP)*

In the same way, patients claimed that most informational materials lack representation of both genders of nurses.

*“I think most informational materials lack representation of male nurses in promotional content for various nursing specialities. It sends a message that certain fields are not as welcoming or suitable for male nurses”.*

*(FGD3-C)*

***Existing unequal opportunities for gender in nursing at the training institution***

NN-HCPs in different FGDs mentioned that certain specialties, skills, or competencies were encouraged or discouraged based on gender. The gender bias is observed when assigning nursing students to clinical rotations.

*“I’ve seen the situation where certain specialities were encouraged or discouraged based on traditional gender roles or stereotypes”.*

*(FGD1-NN-HCP)*

*“I’ve observed biases in assigning clinical rotations during my interactions with nursing education programs. This biased distribution of experiences might unintentionally affect the skill sets and career paths of male and female nurses thus influencing the roles they’re expected to fulfil after graduation”.*

*(FGD3-NN-HCP)*

*“For the students who come to practice, I have noticed that there are instances where certain clinical rotations might have been influenced by gender-based assumptions or stereotypes”.*

*(FGD4-NN-HCP)*

Patients communicated their experiences by which nurses study courses based on their gender.

*“It made me feel like there were certain courses that were off-limits to me based on my gender, which shouldn’t be the case”.*

*(FGD3-C)*

***The bias against male nurse students at the training institution***

Bias is observed in the encouragement of undertaking certain specialties between male and female nursing students. Most nurses in different FGDs reported that some instructors during skill training provide more guidance and detailed information to male nursing students than female nursing students.

*“I also could recall where I noticed instances where certain instructors tended to provide more guidance and opportunities for male nursing students during hands-on training”.*

*(FGD2-N)*

*“In my nursing program, I noticed that during practical demonstrations, instructors sometimes provided more detailed feedback to male students while overlooking similar efforts from us female students”.*

*(FGD2-N)*

*“Back in the school, there were instances where the instructors unintentionally favoured male students during skills assessments. It was understated, but it created a perception that certain skills were characteristically better performed by male nurses”.*

*(FGD1-N)*

*“Certainly. Back in my nursing program, I observed that male students were sometimes given more hands-on experiences”.*

*(FGD1-N)*

*“Also as for me, during my nursing studies, there were occasions where certain practical skills training sessions were assumed to be more suitable for male students”.*

*(FGD3-N)*

Bias in gender in nursing at the training institutions has also been reported, in which male nursing students are more supported and receive exceptional opportunities than their female nursing student counterparts.

*“It felt like there was an assumption that male students needed extra support or encouragement”.*

*(FGD2-N)*

*“I can recall that in my nursing program, I noticed that during simulation exercises, male students were often assigned leadership roles, while female students were more frequently assigned to supporting roles”.*

*(FGD4-N)*

*“As male nursing students, we were often encouraged and praised more for taking on leadership roles during simulations and practical training. It felt like there was an unconscious bias, assuming that we were inherently more capable in leadership positions”.*

*(FGD2-N)*

*“In my nursing program, there were instances where certain clinical opportunities, especially those perceived as more challenging, were more readily given to male students. It seemed like there was an assumption that they were better suited for those experiences”.*

*(FGD1-N)*

*“Just like in roles and responsibility, the training part also has and had its bias during our study days as there were instances during our training where advanced training opportunities were more encouraged and accessible for male nurses. It created a perception that certain areas were more suitable for one gender over another”.*

*(FGD4-N)*

Similarly, NN-HCP identified that male nursing students receive more opportunities than female nursing students do.

*“When back in school I remember the nursing leaders being always male”.*

*(FGD1-NN-HCP)*

Nurses in FGDs said that male nursing students are encouraged to participate in the advanced development of careers, select specialized areas such as critical care, and opt for leadership programs.

*“I have noticed situations where male nursing students received more encouragement to pursue specialized areas such as critical care or leadership roles during our training. It seemed like there was an assumption that these roles were more suitable for male nurses”.*

*(FGD1-N)*

*“Certainly. Back in my nursing program, I observed that male students were sometimes given opportunities in specialized areas during clinical training, reinforcing the idea that certain roles were more suitable for males”.*

*(FGD1-N)*

In the same way, NN-HCPs said that male nursing students were advised to undertake certain specialties, making them unique from female nursing students.

*“Advice or recommendations regarding career paths or specialities might have been influenced by gender stereotypes, potentially limiting the scope of opportunities for some students as the majority of male nurses I know talk of advancing in critical care, mental health, and Operating Theatre Management (OTM) while the majority of the female are into midwifery and pediatric”.*

*(FGD4-NN-HCP)*

On the other hand, patients in different FGDs reported that male nurses are encouraged to pursue certain specialties like critical care and mental health and discouraged from undertaking midwifery and pediatric programs.

*“I have my brother who has studied nursing as first degree and is being encouraged to pursue critical care nursing at an advanced level with the argument that maternity education is for women and it is not good for a male to study the maternity course unless you are studying medicine”.*

*(FGD1-C)*

*“Male nurses in mental health”.*

*(FGD3-C)*

*“I think the thought that male nurses are not suited for courses like maternity or pediatric so maybe they go to other programs”.*

*(FGD4-C)*

Nevertheless, male nurses are assumed to be leaders naturally.

*“There seemed to be an unspoken expectation about the areas male and female nursing students might excel in. For instance, assuming that male students would naturally be leaders”.*

*(FGD2-C)*

*“I can relate to that as in some educational settings, there seemed to be an unspoken expectation male students are always elected as leaders”.*

*(FGD4-C)*

***The bias against female nursing students at training institutions***

Nurses in different FGDs mentioned that the bias of gender in nursing at the training institutions has influenced female nursing students to be directed to certain specialties.

*“I also noticed that the female students were more often directed towards specialities perceived as nurturing or supportive”.*

*(FGD1-N)*

*“Female nurses were directed towards areas perceived as more traditional”.*

*(FGD3-N)*

*“I recall instances during training sessions where we as female nurses were discouraged from pursuing OTM with comments like “it might be too demanding for you.” It felt like there was an underlying bias”.*

*(FGD3-N)*

NN-HCPs in FGDs confirmed the truth, whereby they reported that the majority of female nursing students can be found more in midwifery and pediatric programs.

*“Majority of the females are into midwifery and pediatric”.*

*(FGD4-NN-HCP)*

Nurses in FGDs revealed that females were treated differently during skill acquisition. Female nursing students considered that there were certain skills they should acquire and were assigned to supporting roles during simulation exercises.

*“I recall instances during our training where certain skills were assumed to be more suited for female nurses but that was the time when nursing class had 90% of students as females. I’ll be interested to know the current situation in class”.*

*(FGD3-N)*

*“I can recall that in my nursing program, I noticed that during simulation exercises, female students were more frequently assigned to supporting roles”.*

*(FGD4-N)*

This information is supported by patients who mentioned that female students are more directed to pediatrics and family care programs. Moreover, they are believed to be better at communication and interpersonal skills.

*“I have noticed in pamphlets about nursing careers, a division of roles based on gender. Female nurses are featured in areas like paediatrics and family care”.*

*(FGD3-C)*

***Addressing gender bias at the training institutions***

Many NN-HCPs and patients in different FGDs provided suggestions of what should be done to mitigate the bias of gender in nursing at the training institutions. The suggested strategies are exercising fairness at the nursing training institutions, providing equitable educational opportunities for all aspiring nurses, emphasizing inclusivity and embracing diversity in education, ensuring that all nurses regardless of gender have equal chances to explore various specialties and roles, reassessing and update educational materials and training approaches, and reviewing policies.

### 3.14. Exercising Fairness at Nursing Training Institutions

NN-HCPs have emphasized the benefit of exercising fairness in delivering education to nurses of both genders, as it may minimize or control bias.

*“I tend to believe in the fairness of nursing training”.*

*(FGD1-NN-HCP)*

Patients also mentioned fairness, as it is believed to be a tool to promote balance between genders.

### 3.15. Providing Equitable Educational Opportunities

NN-HCPs have suggested that equal distribution of opportunities in both genders, especially the training and development of careers, is very important.

*“These biases have prompted me to reconsider the importance of providing equitable educational opportunities for all aspiring nurses. Addressing biases in education is vital in ensuring that both male and female nurses receive comprehensive training and exposure to diverse experiences, preparing them equally for their future roles”.*

*(FGD1-NN-HCP)*

*“It’s important to ensure that both male and female nurses have access to a well-rounded education that prepares them for diverse roles without limitations based on gender”.*

*(FGD1-NN-HCP)*

*“All nurses, regardless of gender, receive comprehensive education and exposure to various specialties. It’s about preparing a well-rounded nursing workforce capable of meeting diverse patienti needs”.*

*(FGD2-NN-HCP)*

*“Emphasizes the necessity of equal training opportunities for all aspiring nurses”.*

*(FGD3-NN-HCP)*

*“I think it’s crucial to provide equal opportunities and exposure to diverse experiences, regardless of gender, to prepare nurses for their future roles effectively”.*

*(FGD4-NN-HCP)*

*“It is also essential to challenge and provide equal opportunities and diverse experiences to both male and female nurses, ensuring they’re equipped for a wide range of roles within healthcare for the overall patient care”.*

*(FGD4-NN-HCP)*

*“We need to provide opportunities that foster skill development and prepare nurses of all genders for various roles within the healthcare landscape and this way whether male or female, we will encourage our family members regardless of gender to pursue nursing programs”.*

*(FGD4-NN-HCP)*

Patients, on the other hand, suggested in the same way that every gender in nursing should have the opportunity to pursue any specialty.

*“I love the difference that males should study specific courses and female-specific courses as it feels like everyone brings something valuable to the table because of their gender differences”.*

*(FGD2-C)*

*“Education programs should actively promote a mixture of nursing specialities, ensuring that students feel encouraged to pursue their interests regardless of gender”.*

*(FGD3-C)*

### 3.16. Embracing Diversity in Education

NN-HCPs revealed the benefit of diversity in education, as it promotes confidence among nursing students and helps the implementation of diverse roles

*“There is a need to emphasize inclusivity and diversity in education to shape a future where male and female nurses can confidently pursue diverse roles without gender-related limitations”.*

*(FGD1-NN-HCP)*

### 3.17. Update Educational Materials and Training Approaches and Review Policies

NN-HCPs have shown that the educational materials used in academic institutions need to be updated to accommodate both genders in nursing. Moreover, the training approaches need to reflect the situation of avoiding bias.

*“I think there is a need to reassess and update educational materials and training approaches”.*

*(FGD2-NN-HCP)*

*“The education system should promote a curriculum that challenges gender stereotypes and provides diverse learning experiences is vital in shaping well-rounded nurses for the future”.*

*(FGD2-NN-HCP)*

*“The educational materials and training approaches should be evaluated to ensure they don’t unconsciously reinforce gender categories to prepare future nurses for diverse roles in healthcare”.*

*(FGD3-NN-HCP)*

Consistently, patients delineate the significance of reviewing the existing learning materials to ensure that they match the demand for accommodating both genders in nursing.

*“I think though not sure that the learning materials should be reviewed”.*

*(FGD3-C)*

Furthermore, NN-HCPs insisted that every nurse regardless of gender should have equal opportunity to join any specialty.

*“Ensure that nursing education provides equal opportunities and exposure to various specialties and experiences for all aspiring nurses, regardless of gender”.*

*(FGD3-NN-HCP)*

### 3.18. Changing the Naming

Only patients mentioned the need to change how nurses are being addressed, especially “sisters” since it introduces bias when having male nurses.

*“Look for a better terminology of calling a nurse rather than sister”.*

*(FGD3-C)*

## 4. Discussion

Nurses, non-nurse healthcare providers, and patients participated in different FGDs to respond to the existing bias of gender in nursing in Tanzania. The discussion is based on the following three themes and subthemes.

**①** **Role distribution based on nurse gender, impact, and mitigating approaches for biased role distribution**

In the distribution of roles to male nurses at healthcare facilities, the majority of participants reported that male nurses are assigned to leadership or administrative roles; are more allocated to emergency or critical units having severely sick patients; and are assigned to heavy lifting tasks, technical roles, specialized tasks, and administration of medicine. Furthermore, male nurses receive exceptional opportunities compared to female nurses, like being assigned to more favorable shifts or rotations or assigned to participate in workshops or seminars. Meanwhile, female nurses are assigned to documentation tasks, bedside care, administrative tasks of being in charge of female units, taking vital signs, bed making, bathing patients, delivering health education to patients, communication-demanding tasks, and are allocated to pediatric wards, maternity wards, and other units with female patients.

All these roles that male nurses are assigned to carry out have elements of what communities socially believe men are capable of doing. For example, the communities assume men are masculine and are able to perform physically demanding activities, which is consistent with a previous study [42]. That is why male nurses can be found involved in physically demanding tasks or units because they are trusted in their power to lift or carry heavy weights. They are also assigned to leadership roles because men are socially entrusted to have the abilities of decision-making and critical thinking, which is supported by previous articles that men are expected to figure out things without any assistance [43]. This stereotype makes men privileged in obtaining high governmental positions to lead others [44].

Similarly to female nurses, their distributed roles look simple and easy to carry out, which might be because it is socially believed that women have caring elements and are supposed to do simple tasks that demand little critical thinking and decisions. This is supported by previous information reported that society expects women to take care of the children, cook, and clean the home [45]. In contrast, even though the roles mentioned to be assigned to female nurses look simple, they are not, demanding individual competence and ability to decide. For instance, bedmaking and taking vital signs require somebody to understand the principles of performing those particular procedures. Therefore, role distributors based on gender need to understand that any nursing procedure should not be considered simple because it requires the application of principles and interpretations. This is supported by a previous study that reported that bedmaking requires technical and practical skills and consideration should be given to issues of safety, moving and handling, and infection control practices [46].

In this current study, the distribution of roles to male and female nurses is not based on professionalism, individual abilities, or competence, but rather the adoption of community beliefs and culture that men are masculine and women have caring qualities.

It was found that the biased role distribution for genders in nursing hurts all nurses, especially exacerbating frustration, creating a sense of inequalities, and affecting work motivation. This might be because nurses of any gender would expect to be assigned to a certain role or allocated to a certain unit due to his/her competence, but if it happens otherwise, they would feel under-graded and hence lack motivation. A previous study revealed that the biased role distribution exacerbates job dissatisfaction [47] and hinders female nurses from growing their careers [48].

Participants suggested different strategies to avoid bias in role distribution, whereby most of them insisted on transparency during role distribution, establishing role qualifications, and granting equal opportunities to both genders. Based on these three approaches, an environment can be created where everyone has a chance to be allocated to a certain role. If leaders can consider them equal, the bias may be avoided. Other mentioned strategies include having objectives, criteria, and guidelines; making resources available to both genders; leaders’ initiatives; training; open communication; and fairness.

**②** **Different ways of addressing challenges in gender in nursing diversity**

The majority of participants emphasized that training and development are among the optimal solutions for mitigating the challenge of diversity in nursing. Training refers to a short-term program to improve the work skills of an individual, while development is a long-term program for career development. All of them enhance individual competencies for effective performance. When both genders have the required competencies, they have an equal chance to be assigned to a certain role. This is supported by a previous study indicating that both genders should establish their career through development [22]. Meanwhile, open communication has been reported as a means to control the challenges of the diversity of gender in nursing. This is because when there is transparency, no one may raise a concern about inequality, and they can thus become satisfied. Open communication needs to be exercised when assigning an individual to a certain opportunity or allocating to a particular role. Nevertheless, recognizing and valuing team member skills is mentioned by the majority as it encourages a culture of professionalism, and everyone feels respected. Moreover, leaders’ efforts to prevent and control challenges seem to be vital. They should be the role model to demonstrate that gender in nursing is not important, but rather, professionalism is more considered. This can come true if leaders become conscious and sensitive to competencies and not gender. Additionally, leaders should be responsible for promoting awareness of subordinates to make them aware of the consequences of bias in gender in nursing. Since leaders deal with management processes and sometimes engage in strategic development, they are in a good position to mitigate the challenges of gender in nursing. This is consistent with another previous study that mentioned that nursing leaders should offer long-term strategic solutions [49]. Other approaches include mutual respect, contribution, value of the diversity of gender in nursing, collaboration, prioritizing the needs and preferences of patients above all else, and equitable treatment for all team members.

**③** **Gender in nursing biases at the training institutions**

The bias of gender in nursing is present at training institutions. It is more observed during the learning process, as men are encouraged to focus on masculine-related programs while women are encouraged to caring-related programs. Since nursing has historically been considered a feminine profession, men align themselves to challenge the notion by opting for a program that will still show their masculinity.

During the learning process, the materials and scenarios focus more on females than males. This can be perpetuated by the history of nursing, which referred to nurses as sisters, evidenced by some of the outdated materials addressing all nurses regardless of gender as sisters. This is supported by a previous study that mentioned that some instructional materials such as lecture notes, case studies, or pictures in PowerPoint slides insinuate a stereotype [50].

Moreover, the facilitators show more favor to male nursing students to acquire skills than they do to female nursing students. This kind of facilitator still holds the elements of traditional nursing. Participants reported how the bias in training institutions can be prevented and controlled by providing equitable educational opportunities for all aspiring nurses. Most of the participants feel that any program can be pursued by any nursing student regardless of gender. If everyone has a chance of attending a desired program, they will be able to obtain the required competencies. Moreover, the teaching strategies and instructional materials should be customized to reflect a non-biased gender, which is consistent with previous documentation by Strong [51]. Exercising fairness at nursing training institutions has been reported as a way to mitigate bias because when both genders are equally treated, the teaching process will take consideration of both genders, and each will have an equitable chance for available opportunities, making learning more effective. This conforms to a previous study that stated that teachers should provide equal opportunities to nursing students [52]. Other approaches emphasize embracing diversity in education, ensuring that all nurses regardless of gender have equal chances to explore various specialties and roles, reassessing and updating educational materials and training approaches, and reviewing policies.

## 5. Conclusions

The bias and stereotypes about gender in nursing still exist in the clinical areas and training institutions. Exercising professionalism in both settings remains a vital aspect of reducing bias. Nevertheless, role distribution should not be dominated by social roles of men and women in the community but rather should be based on competence and individual abilities. The role distributors based on gender hold non-professional thinking when considering some nursing procedures to be simple and to be assigned to female nurses, without knowing that those procedures require one’s critical thinking skills, interpretation skills, and decision-making. Having leaders in nursing who recognize professionalism and who have good clinical competence backgrounds would help to address these issues.

## Figures and Tables

**Table 1:** Study characteristics.

Population	Facilities	Interviewee in FGD	Sex	Professional	Experience
**Nurses**	HOSPITAL 1	P1	Female	Nurse	10 years
		P2	Male	Nurse	-
		P3	Female	Nurse	7 years
		P4	Male	Nurse	-
		P5	Female	Nurse	9 years
	HOSPITAL 2	P1	Male	Nurse	5 years
		P2	Male	Nurse	8 years
		P3	Male	Nurse	2 years
		P4	Male	Nurse	4 years
		P5	Female	Nurse	-
		P6	Female	Nurse	-
	HOSPITAL 3	P1	Male	Nurse	Over 10 years
		P2	Female	Nurse	8 years
		P3	Male	Nurse	5 years
		P4	Female	Nurse	-
		P5	Female	Nurse	-
	HOSPITAL 4	P1	Male	Nurse	6 years
		P2	Female	Nurse	5 years
		P3	Female	Nurse	Over 10 years
		P4	Female	Nurse	-
		P5	Male	Nurse	-
**Non-Nurse Healthcare Providers**	HOSPITAL 1	P1	Male	Medical doctor	Over 15 years
		P2	Male	Pharmacist	Over 8 years
		P3	Male	Lab technician	7 years
		P4	Female	Medical doctor	Over 5 years
	HOSPITAL 2	P1	Male	Medicine (Physician)	-
		P2	Female	Pharmacist	-
		P3	Female	Lab technician	-
		P4	Female	Medicine (Physician)	-
	HOSPITAL 3	P1	Female	Pharmacist	7 years
		P2	Male	Medical laboratory scientist	-
		P3	Female	Medical doctor	6 years
		P4	Male	Medical doctor	5 years
	HOSPITAL 4	P1	Male	Senior medical laboratory scientist	22 years
		P2	Male	Medical doctor	15 years
		P3	Female	Pharmacist	-
		P4	Female	-	10 years
**Patients**	HOSPITAL 1	P1	Female		
		P2	Male		
		P3	Female		
		P4	Female		
		P5	Male		
		P6	Female		
	HOSPITAL 2	P1	Female		
		P2	Female		
		P3	Female		
		P4	Male		
		P5	Male		
		P6	Male		
	HOSPITAL 3	P1	Male		
		P2	Female		
		P3	Female		
		P4	Male		
		P5	Male		
		P6	Female		
	HOSPITAL 4	P1	Male		
		P2	Female		
		P3	Female		
		P4	Female		
		P5	Female		

**Table 2:** The summary of themes and subthemes.

S/NO	Subthemes	Themes
1	Distribution of roles to male nurses at healthcare facilities	① Role distribution based on gender in nursing, impact and mitigating approaches for biased role distribution
2	Distribution of roles to female nurses at healthcare facilities	
3	Impact of biased role distribution on gender in nursing	
4	Approaches to mitigate biased role distribution between genders in nursing	
1	Addressing bias in gender in nursing diversity through training and mentorship	② Different ways of addressing challenges in gender in nursing diversity
2	Addressing bias in gender in nursing diversity through leaders’ efforts	
3	Addressing bias in gender in nursing diversity through open communication	
4	Addressing bias in gender in nursing diversity through mutual respect	
5	Addressing bias in gender in nursing diversity by recognizing and valuing team member skills, and contribution	
6	Value the diversity of gender in nursing	
7	Addressing bias in gender in nursing diversity through collaboration	
8	Prioritizing the needs and preferences of clients above all else	
9	Equitable treatment for all team members	
1	Teaching methods encouraging gender in nursing bias at training institutions	③ Gender in nursing biases at the training institutions
2	Existing unequal opportunities for gender in nursing at the training institution	
3	The bias against male nurse students at the training institution	
4	The bias against female nurse students at training institutions	
5	Addressing gender bias at the training institutions	

## Data Availability

Data availability statements are available in.

## Public Involvement Statement

Healthcare providers and non-healthcare providers were involved during data collection by responding to the questionnaire.

## Guidelines and Standards Statement

COREQ reporting guidelines for qualitative research were used.
